# The shared genetic architecture of suicidal behaviour and psychiatric disorders: A genomic structural equation modelling study

**DOI:** 10.3389/fgene.2023.1083969

**Published:** 2023-03-07

**Authors:** Tahira Kootbodien, Leslie London, Lorna J. Martin, Joel Defo, Raj Ramesar

**Affiliations:** ^1^ UCT/MRC Genomic and Precision Medicine Research Unit, Division of Human Genetics, Department of Pathology, Institute for Infectious Diseases and Molecular Medicine, University of Cape Town and Affiliated Hospitals, Cape Town, South Africa; ^2^ School of Public Health and Family Medicine, University of Cape Town, Cape Town, South Africa; ^3^ Division of Forensic Medicine and Toxicology, Department of Pathology, University of Cape Town, Cape Town, South Africa

**Keywords:** psychiastric disorders, genomic structural equation modeling, GWAS-genome-wide association study, suicidal behavior (SB), genetic correlation, mendelian randomization

## Abstract

**Background:** Suicidal behaviour (SB) refers to behaviours, ranging from non-fatal suicidal behaviour, such as suicidal ideation and attempt, to completed suicide. Despite recent advancements in genomic technology and statistical methods, it is unclear to what extent the spectrum of suicidal behaviour is explained by shared genetic aetiology.

**Methods:** We identified nine genome-wide association statistics of suicidal behaviour (sample sizes, *n*, ranging from 62,648 to 125,844), ten psychiatric traits [*n* up to 386,533] and collectively, nine summary datasets of anthropometric, behavioural and socioeconomic-related traits [*n* ranging from 58,610 to 941,280]. We calculated the genetic correlation among these traits and modelled this using genomic structural equation modelling, identified shared biological processes and pathways between suicidal behaviour and psychiatric disorders and evaluated potential causal associations using Mendelian randomisation.

**Results:** Among populations of European ancestry, we observed strong positive genetic correlations between suicide ideation, attempt and self-harm (rg range, 0.71–1.09) and moderate to strong genetic correlations between suicidal behaviour traits and a range of psychiatric disorders, most notably, major depression disorder (rg = 0.86, *p* = 1.62 × 10^−36^). Multivariate analysis revealed a common factor structure for suicidal behaviour traits, major depression, attention deficit hyperactivity disorder (ADHD) and alcohol use disorder. The derived common factor explained 38.7% of the shared variance across the traits. We identified 2,951 genes and 98 sub-network hub genes associated with the common factor, including pathways associated with developmental biology, signal transduction and RNA degradation. We found suggestive evidence for the protective effects of higher household income level on suicide attempt [OR = 0.55 (0.44–0.70), *p* = 1.29 × 10^−5^] and while further investigation is needed, a nominal significant effect of smoking on suicide attempt [OR = 1.24 (1.04–1.44), *p* = 0.026].

**Conclusion:** Our findings provide evidence of shared aetiology between suicidal behaviour and psychiatric disorders and indicate potential common molecular mechanisms contributing to the overlapping pathophysiology. These findings provide a better understanding of the complex genetic architecture of suicidal behaviour and have implications for the prevention and treatment of suicidal behaviour.

## 1 Introduction

Suicidal behaviour is a major public health concern. It is estimated that approximately 700,000 individuals die by suicide every year; with a global suicide rate of 9.0 per 100,000 population (WHO, 2021). According to the Global Burden of Diseases, Injury and Risk Factors Study (GBD 2019), suicidal behaviour was estimated to be responsible for nearly 34.1 million disability-adjusted life years (DALYs) globally in 2019, of which the majority occurred in those aged 10–49 years (Vos et al., 2020; IHME, 2022). Worldwide, suicide is the fourth leading cause of death in 15–29-year-olds (WHO, 2021).

Suicidal behaviour is a broad and complex term used to describe suicidal thoughts and a range of self-injurious behaviour involving intent to die (suicide attempt, self-harm and death) (Posner et al., 2007). Attempted suicide is considered an important risk factor for subsequent suicide (Hawton et al., 2015) and is 25–30 times more common than completed suicide (Schmidtke et al., 1996). The risk of death after re-attempting suicide is higher in the first year after an attempted suicide, with 2.3% of subsequent re-attempts resulting in death (Bostwick et al., 2016). While there has been substantial evidence that individuals with suicidal thoughts are at increased risk for later or subsequent suicidal ideation, attempts and death (Ribeiro et al., 2016), most individuals may never act on their thoughts (Nock et al., 2009). Previous research has explored the progression of suicidal thoughts to suicidal behaviour by applying various theories of suicide (Nock et al., 2013; May and Klonsky, 2016). Several studies have highlighted that the risk factors involved in the development of suicidal ideation are different from those who transition to suicide attempts (Nock et al., 2008; Klonsky et al., 2017). Given that suicidal behaviour is an outcome that results from many factors, and the spectrum of behaviour may reflect a continuum of suicide risk (Sveticic and De Leo, 2012), it is important to understand the pathways leading to completed suicide. Understanding the pathways from less to severe suicidal behaviour is relevant as it provides additional opportunities for suicide prevention at different stages of risk.

Suicidal behaviour is partly genetic, with moderate heritability estimates ranging from 38%–55% in adoption, twin and family studies (reviewed by Brent and Mann, 2005; Voracek and Loibl, 2007; Brent and Melhem, 2008) and 17% and 36% for suicide attempt and ideation respectively, when controlling for psychiatric illness (Fu et al., 2002). It is well established that psychiatric comorbidities play an important role in the development of suicide, as approximately 90% of individuals who die by suicide have been reported to have a diagnosed psychiatric disorder (Arsenault-Lapierre et al., 2004). Psychiatric disorders such as depression, bipolar mood disorders, schizophrenia, post-traumatic stress disorder, substance use and eating disorders have been associated with suicide (Nock et al., 2010). Suicide has also been linked to attention deficit hyperactivity disorder (ADHD) (Giupponi et al., 2018) and sleep disorders (Bernert et al., 2015). Other risk factors include smoking (Poorolajal and Darvishi, 2016), poverty (Iemmi et al., 2016) and educational disparities (Lorant et al., 2021). Moreover, suicidal behaviour is also included as part of the diagnostic criteria for major depression and bipolar disorders (Fehling and Selby, 2021), meaning that suicide or suicidal behaviour is considered to be a symptom of these disorders. Studies have shown that many psychiatric disorders share a common set of genetic factors (Caspi et al., 2014; Lee et al., 2019; Allegrini et al., 2020). The shared genetic liability captured onto a single dimension called the “*p* factor,” may explain why so many psychiatric disorders are comorbid (Plomin et al., 2016). The theoretical concept, the *p* factor, suggests that components of the underlying pathology of psychiatric disorders may be shared across several (if not all) psychiatric disorders. This framework was further supported by Allegrini and others who reported that the *p* factor remained stable across childhood and adolescence over a life course, suggesting that the shared genetic influences of psychiatric disorders in childhood is also linked to the development of adult psychiatric disorders (Allegrini et al., 2020). While still in its infancy, research findings from investigations on the *p* factor suggest that the comorbidity of several psychiatric disorders may be explained by a common or shared genetic pathway/s.

While genome-wide association studies (GWAS) have continued to explain only a small proportion of the heritability of suicidal behaviour, the increase in the availability of data from studies with larger sample sizes over the last few years, has expanded the scope of available statistical methods to improve the understanding of suicide burden (Wang et al., 2011; Loos, 2020). One such method, the analyses of single nucleotide polymorphism (SNP)-based genetic correlations using genomic structural equation modelling (GenomicSEM), has identified patterns of shared genetic architecture across many psychiatric disorders (Grotzinger et al., 2019; Lee et al., 2019). In this study, we proposed a common factor model that represents an extension of the general psychopathology or genomic “*p* factor” that includes suicidal behaviour using Genomic SEM. We performed a gene/pathway-specific meta-analysis and functional enrichment to identify a set of genes at the subnetwork level significantly associated with the common factor. We applied Mendelian randomisation to identify potentially pleiotropic and causal relationships between modifiable risk factors and suicidal behaviour and further highlighted potential drugs interacting with the subnetwork genes that may be targeted for future drug development.

## 2 Materials and methods

### 2.1 Description of GWAS summary data

This study was conducted using 28 publicly available genome-wide association studies (GWAS) summary data generated by previous studies. We identified nine SB traits, ten psychiatric traits, and five behavioural and two anthropometric and socioeconomic-related variables, respectively (Table 1, web links for downloading data provided). Population ancestry was grouped as European if the study population was described as “Caucasian” or “White” by the author and as East Asian if the study population was described as “Japanese” or “Han Chinese”. Suicidal behaviour datasets were derived from GWAS samples of both sexes of European ancestry for suicidal ideation (*n* = 4), suicide attempt (*n* = 2), and self-harm (*n* = 2), and summary statistics of completed suicide in an East Asian population.

**TABLE 1:** Data sources and description of suicidal behaviour, psychiatric, behavioural and socioeconomic GWAS datasets.

Phenotype/Reference	Ancestry	Consortium/Source	Sample size	Variable	#Cases	#Controls	Web links/URLs
***Suicidal behaviour***							
*Suicidal ideation*							
Recent thoughts of suicide or self-harm	EUR	United Kingdom Biobank/Neale lab	125,844	Continuous	NA	NA	https://www.nealelab.is/uk-biobank
Thought life not worth living (TLNWL)	EUR	United Kingdom Biobank/Neale lab	117,291	Continuous	NA	NA	https://www.nealelab.is/uk-biobank
Thoughts of death during worst depression	EUR	United Kingdom Biobank/Neale lab	62,648	Binary	32,630	30,018	https://www.nealelab.is/uk-biobank
Ever contemplated self-harm (ECSH)	EUR	United Kingdom Biobank/Neale lab	117,610	Continuous	NA	NA	https://www.nealelab.is/uk-biobank
*Suicide attempt*							
Attempted suicide (Erlangsen et al., 2018) (SA)	EUR	iPSYCH	50,264	Binary	6,024	44,240	https://ipsych.dk/en/research/downloads/
Ever attempted suicide	EUR	United Kingdom Biobank/Neale lab	4,933	Binary	2.658	2.275	https://www.nealelab.is/uk-biobank
*Self-harm*							
Ever self-harmed (ESH)	EUR	United Kingdom Biobank/Neale lab	117,733	Binary	5,099	112,634	https://www.nealelab.is/uk-biobank
Seeking mental health services	EUR	United Kingdom Biobank/Neale lab	117,733	Binary	1,693	116,040	https://www.nealelab.is/uk-biobank
*Completed suicide*							
Suicide death (Otsuka et al., 2019)	EAS		14,795	Binary	746	14,049	https://humandbs.biosciencedbc.jp/hum0196-v1
***Psychiatric traits***							
Schizophrenia (Pardinas et al., 2018)	EUR	Clozuk + PGC2	105,318	Binary	40,675	64,643	https://walters.psycm.cf.ac.uk/
Schizophrenia (Lam et al., 2019)	EAS	PGC	58,140	Binary	22,778	35,362	https://www.med.unc.edu/pgc/download-results/
Bipolar Disorder (Stahl et al., 2019)	EUR	PGC2 BD	51,710	Binary	20,352	31,358	https://www.med.unc.edu/pgc/download-results/
MDD (Giankopolou et al., 2021)	EAS	PGC	194,548	Binary	15,771	178,777	https://www.med.unc.edu/pgc/download-results/
MDD (Wray et al., 2018)	EUR	PGC	480,359	Binary	135,458	344,901	https://www.med.unc.edu/pgc/download-results/
Anorexia Nervosa (Duncan et al., 2017)	EUR	PGC	14,477	Binary	3,495	10,982	https://www.med.unc.edu/pgc/download-results/
PTSD (Duncan et al., 2018)	EUR	PGC	9,954	Binary	2,489	7,465	https://www.med.unc.edu/pgc/download-results/
ADHD (Demontis et al., 2018)	EUR	PGC	53,293	Binary	19,099	34,194	https://www.med.unc.edu/pgc/download-results/
Alcohol use disorder (Walters et al., 2018)	EUR	PGC	38,686	Binary	10,206	28,480	https://www.med.unc.edu/pgc/download-results/
Insomnia (Jansen et al., 2019)	EUR	United Kingdom Biobank	386,533	Binary	109,402	277,131	https://ctg.cncr.nl/software/summary_statistics
***Behavioural traits***							
Drinks per week (Liu et al., 2019)	EUR	GSCAN	941,280	Continuous	NA	NA	https://conservancy.umn.edu/handle/11299/201564
Drinks per week (Matoba et al., 2020)	EA	GWAS catalogue	58,610	Continuous	NA	NA	https://www.ebi.ac.uk/gwas/downloads/summary-statistics/
Cigarettes per day (Liu et al., 2019)	EUR	GSCAN	337,334	Continuous	NA	NA	https://conservancy.umn.edu/handle/11299/201564
Cigarettes per day (Matoba et al., 2019)	EA	BBJ	72,655	Continuous	NA	NA	http://jenger.riken.jp/en/result
Smoking (ever vs never) Kanai et al., 2021	EA	BBJ	176,166	Binary	88,277	87,889	https://pheweb.jp/pheno/Smoking_Ever_Never
***Anthropometric traits***							
Body mass index (Locke et al., 2015)	EUR	GIANT consortium	322,154	Continuous	NA	NA	https://www.nealelab.is/uk-biobank
Body mass index (Sakau & Kanai et al., 2021)	EA	BBJ	163,835	Continuous	NA	NA	https://pheweb.jp/pheno/BMI
***Socioeconomic traits***							
Household Income (Hill et al., 2016)	EUR	United Kingdom Biobank	112,151	Continuous	NA	NA	http://www.ccace.ed.ac.uk/node/335
Education in years (Okbay et al., 2016)	EUR	SSGAC	293,723	Continuous	NA	NA	https://thessgac.com/papers/

Briefly, the self-report measures of suicide ideation and self-harm were derived from GWAS studies of the United Kingdom Biobank (UKB) population (sample sizes ranged from 62,648 to 125,844) and accessed from the Neale lab (see weblinks/URLs, Table 1). Data within the UKB are structured in datasets and identified using field codes. Suicide ideation measures included recent (i.e., over the last 2 weeks) thoughts of suicide or self-harm (UKB field 20513); thoughts that life was not worth living (TLNWL, UKB field 20479); ever contemplated self-harm (ECSH, UKB field 20485), and having thoughts of death during worst of depression (UKB field 20437). Information on suicide ideation was obtained from three questions in the United Kingdom Biobank: (Recent thoughts of self-harm) “Over the last 2 weeks, how often have you been bothered by any of the following problems?; (TLNWL) “Many people have thoughts that life is not worth living. Have you felt that way?” and (ECSH) “Have you contemplated harming yourself (for example, by cutting, biting, hitting yourself or taking an overdose)?”. The first two questions have three options: “no,” “yes, once” and “yes, more than once”. ECSH have four options: “not at all,” “several days,” “more than half the days” and “nearly every day”. Two datasets of self-reported self-harm include ever self-harmed (ESH, field 20480, *n* = 117,610) and attempted self-harm and needed hospital treatment (UKB field 20554, *n* = 117,733). Attempted suicide datasets were obtained from the United Kingdom Biobank study, a self-report measure indicating having ever attempted suicide (UKB field 20483; *n* = 4,933) and attempted suicide cases (*n* = 6,024) and controls (*n* = 44,240) from a GWAS study from the Lundbeck Foundation Initiative for Integrative Psychiatric Research (iPSYCH) (*n* = 50,254).

GWAS summary statistics were identified (Table 1) for psychiatric traits among European populations (*n* = 7; schizophrenia, bipolar disorder, major depression disorder, anorexia nervosa, PTSD, ADHD and insomnia) and East Asian populations (*n* = 2, schizophrenia and major depressive disorder). Behavioural traits included the average number of drinks per week and smoking habits among individuals of European and East Asian ancestry. Drinks per week (DPW), defined as the average number of drinks a participant reported drinking each week, aggregated across all types of alcohol, was examined in a combined approach with the GSCAN consortium and United Kingdom Biobank (UKB) (Liu et al., 2019) (*N* = 941,280), while cigarettes per day were defined as the average number of cigarettes smoked per day, either as a current or former smoker (Liu et al., 2019) (*n* = 337,334). Summary-level data was obtained for socioeconomic-related traits, i.e., household monthly income from United Kingdom Biobank (Hill et al., 2016) and education achievement, measured in school years (Okbay et al., 2016). The summary datasets included in this study are in the public domain and contain de-identified and anonymised data; thus, ethical approval from an institutional review board was not required for this study.

### 2.2 Data formatting, SNP-based heritability and genetic correlation estimation

#### Data formatting

Datasets were formatted according to requirements for linkage disequilibrium score regression (LDSC) and genomic structural equation modelling (SEM) (Bulik-Sullivan et al., 2015; Grotzinger et al., 2019). We obtained publicly available pre-computed linkage disequilibrium (LD) scores and weights of the 1,000 Genomes European and East Asian reference (https://data.broadinstitute.org/alkesgroup/LDSCORE/). GWAS summary statistics were filtered for SNPs included in HapMap3 to reduce the likelihood of bias induced by poor imputation quality. SNPs were excluded if minor allele frequency (MAF) < 1% and information (INFO) scores <0.9 or if they were located in the human major histocompatibility complex (MHC) region. Datasets without a marker name (rsID) were annotated using ANNOVAR software, with avsnp142, an abbreviated version of dbSNP 142 with left-normalization, on human genome build hg19 (Wang et al., 2010).

#### SNP-based heritability

SNP-based heritability estimates and pairwise genetic correlation were calculated for each dataset using LDSC software (https://github.com/bulik/ldsc) (Bulik-Sullivan et al., 2015). SNP-based heritability is the proportion (that ranges from 0 to 1) of variance of the phenotype that is attributable to all common SNPs used in a GWAS. Heritability estimates are presented in Table 2 and expressed on the observed scale. Lower heritability estimates with larger standard errors relative to the estimate indicated that there was not enough power to detect the SNP-based heritability estimate based on available datasets. Lower heritability estimates with larger standard errors relative to the estimate indicated larger uncertainty in the SNP-based heritability estimate. The genomic inflation factor (or lambda genomic control factor, λGC) compares the median of the resulting chi-squared statistics (χ^2^) divided by the expected median of the chi-squared distribution and was used to assess systematic bias or genomic inflation present in the GWAS summary data due to population stratification. A λGC estimate of around 1, indicates no systematic bias. An LDSC intercept near one indicates little or no confounding and larger than 1.3 indicates that the results might be affected by confounding bias (Bulik-Sullivan et al., 2015). SNP intercepts indicated no confounding bias in this study. The results were visualised using the corrplot package in R (R Core Team, 2017) and the correlation dot plot in SRplot (http://www.bioinformatics.com.cn/srplot). We retained four [Thought life is not worth living (TLNWL), Ever contemplated self-harm (ECSH), Attempted suicide (SA), Ever self-harmed (ESH)] of the nine suicidal behaviour GWAS summary data with genetic correlation estimates with heritability z-scores above 4, as scores below 4 do not produce reliable estimates (Bulik-Sullivan et al., 2015; Grotzinger et al., 2019).

**TABLE 2:** Results of univariate SNP-based heritability estimates using LD-score regression of predicted probability of suicidal behaviour (SB), psychiatric disorders and education in years and income.

Phenotype/Reference	Ancestry	# of SNPs	SNP-based heritability (SE)	z-score	*p*	Mean χ2	λ GC	Intercept (SE)^a^
***Suicidal behaviour (SB)***								
*Suicidal ideation*								
Recent thoughts of suicide or self-harm	EUR	9,561,902	0.0143 (0.0038)	3.76	8.50 × 10^−5^	1.0402	1.0426	1.0067 (0.0064)
Thought life not worth living (TLNWL)	EUR	1,096,648	0.0735 (0.0054)	13.61	1.74 × 10^−42^	1.1788	1.1578	1.0087 (0.0072)
Thoughts of death	EUR	13,559,508	0.0246 (0.0071)	3.46	0.0003	1.0314	1.0309	1.0012 (0.0065)
Ever contemplated self-harm (ECSH)	EUR	11,386,518	0.0427 (0.0051)	8.38	2.65 × 10^−17^	1.1190	1.1093	1.0206 (0.0068)
*Suicidal attempt*								
Attempted suicide (SA)	EUR	11,601,089	0.0799 (0.0123)	6.49	4.29 × 10^−11^	1.1107	1.0988	1.0225 (0.0092)
Ever attempted suicide	EUR	10,941,854	0.1461 (0.0892)	1.64	0.0505	1.0085	1.0061	0.9946 (0.0062)
*Completed suicide*								
Completed suicide (Otsuka et al., 2019)	EA	8,381,404	0.0776 (0.0303)	2.56	0.0052	1.0756	1.0741	1.0514 (0.0075)
*Self-harm*								
Ever self-harmed (ESH)	EUR	12,075,154	0.0217 (0.0044)	4.93	4.11 × 10^−7^	1.0613	1.0536	1.0107 (0.0066)
SH needing hospital treatment	EUR	10,169,094	0.0129 (0.0038)	3.39	0.0004	1.0364	1.0410	1.0065 (0.0062)
***Psychiatric disorders***								
Schizophrenia (Pardinas et al., 2018)	EUR	1,153,380	0.4100 (0.0138)	29.71	2.85 × 10^−194^	1.9325	1.6822	1.0702 (0.0113)
Schizophrenia (Lam et al., 2019)	EA	10,694,924	0.3784 (0.0222)	17.05	1.75 × 10^−65^	1.3100	1.2464	1.0002 (0.0096)
Bipolar Disorder (Stahl et al., 2019)	EUR	1,184,385	0.3872 (0.0190)	20.39	1.03 × 10^−92^	1.3670	1.3061	1.0189 (0.0081)
MDD (Wray et al., 2018)	EUR	1,081,131	0.0774 (0.0047)	15.74	9.29 × 10^−55^	1.2659	1.2365	0.9954 (0.0092)
MDD (Giankopolou et al., 2021)	EA	7,440,942	0.0080 (0.0022)	3.64	0.0001	1.0419	1.0345	1.0093 (0.0065)
Anorexia nervosa (Duncan et al., 2017)	EUR	10,120,601	0.2403 (0.0382)	6.29	1.59 × 10^−10^	1.0793	1.0772	1.0089 (0.0095)
PTSD (Duncan et al., 2018)	EUR	13,206,098	0.0464 (0.0205)	2.26	0.0112	1.0127	1.0165	0.9939 (0.0059)
ADHD (Demontis et al., 2018)	EUR	8,047,421	0.2268 (0.0145)	15.64	1.94 × 10^−55^	1.2966	1.2531	1.0336 (0.0102)
Alcohol use disorder (Walters et al., 2018)	EUR	9,271,144	0.0952 (0.0199)	4.78	8.76 × 10^−7^	1.0601	1.0588	1.0182 (0.0063)
Insomnia (Jansen et al., 2019)	EUR	1,117,678	0.0456 (0.0019)	24.00	1.39 × 10^−127^	1.3617	1.3061	1.0140 (0.0078)
***Behavioural traits***								
Drinks per week (Liu et al., 2019)	EUR	11,916,706	0.0485 (0.0021)	23.09	2.92 × 10^−118^	1.4472	1.3169	0.9267 (0.0084)
Drinks per week (Matoba et al., 2020)	EA	5,961,480	0.0731 (0.0420)	1.740	0.0819	1.0892	1.0225	1.0009 (0.0080)
Cigarettes per day (Liu et al., 2019)	EUR	12,003,613	0.0724 (0.0068)	10.65	8.72 × 10^−27^	1.3301	1.2201	0.9595 (0.0095)
Cigarettes per day (Matoba et al., 2019)	EA	5,925,778	0.0669 (0.0116)	5.76	4.46 × 10^−9^	1.1059	1.0957	1.0096 (0.0088)
Smoking (ever vs never) Kanai et al., 2019	EA	13,531,752	0.0290 (0.0042)	6.90	2.60 × 10^−12^	1.1092	1.0975	1.0024 (0.0081)
***Anthropometric traits***								
Body mass index (Locke et al., 2015)	EUR	2,554,637	0.1297 (0.0056)	23.16	5.76 × 10^−119^	1.2603	1.0772	0.6729 (0.0079)
Body mass index (Sakau & Kanai et al., 2021)	EA	13,236,464	0.1772 (0.0078)	22.72	1.42 × 10^−114^	1.6636	1.4926	1.0740 (0.0188)
***Socioeconomic-related traits***								
Income (Hill et al., 2016)	EUR	1,217,311	0.0599 (0.0056)	10.70	5.09 × 10^−27^	1.1613	1.1428	1.0290 (0.0071)
Education years (Okbay et al., 2016)	EUR	8,146,840	0.1108 (0.0037)	29.95	2.19 × 10^−197^	1.6445	1.4745	0.9377 (0.0092)

#### Genetic correlation

We calculated pairwise genetic correlation, i.e., the standardised proportion of the variance shared by the phenotypes that can be attributed to genetic factors, using LDSC, a method that is not biased by sample overlap. Correlations are reported as the coefficient ±standard error. To note, the LDSC estimator is unbounded and can produce genetic correlation estimates outside of −1 to 1 due to sampling variation. (See https://groups.google.com/g/ldsc_users/c/3jtyM4mmTGs). Genetic correlations were corrected for multiple testing based on the total number of correlations by applying a Bonferroni corrected threshold of *p* < 0.05/52, corrected for four suicidal behaviour traits x 13 psychiatric, sociodemographic and behavioural traits, 9.615 × 10^−4^ for GWAS studies of European ancestry and seven East Asian studies of completed suicide and psychiatric disorders (*p* < 0.05/7 = 0.007).

### 2.3 Genomic structural equation modelling

We performed exploratory and confirmatory factor analysis using the R-package Genomic Structural Equation Modelling (GenomicSEM) (Grotzinger et al., 2019). This method performs structural equation modelling using GWAS summary statistics, allowing us to explore the genetic factor structure of the suicidal behaviour traits and psychiatric traits. We used the Genomic SEM’s multivariable LD score regression method to estimate the genetic covariance matrix (S) and sampling covariance matrix (V) for all traits. All SNPs were standardized using the sumstats function in Genomic SEM (Grotzinger et al., 2019). We fit models using genetic covariance and sampling covariance matrices to examine the genome-wide factor structure of the data. We derived a single genomic factor or common factor containing genome-wide factor loadings representing each SNP contribution to the shared liability of suicidal behaviour and psychiatric disorders. Because “Ever contemplated self-harm” (ECSH) was highly correlated with “Thought life not worth living” (TLNWL), we retained the suicidal ideation trait with the highest SNP heritability z-score i.e., TLNWL (z-score = 13.61). Next, we performed an exploratory factor analysis of the S matrix with one, two and three factors using promax rotation in the R package factanal to guide the construction of a follow-up model. Standardised loadings of more than 0.4 were retained. We assessed model fit by comparing recommended test results and cut-offs; a good fit is indicated by a Comparative Fit Index (CFI) ≥ 0.95, Standardized Room Mean Square Residual (SRMR) ≤ 0.05 and lower AIC values indicate a better fit (Grotzinger et al., 2019). We extended genomicSEM to examine the relationship between the common factor and socioeconomic-related (education years and household income) and behavioural risk factors (smoking and average drinks per week). Because of the low SNP-based heritability z-scores (z-scores <4) observed among populations of East Asian ancestry, genomic SEM analyses were conducted on datasets of European ancestry populations only.

### 2.4 Gene and pathway-specific meta-analysis

We performed gene/pathway-specific meta-analysis by combining the effect size of multiple SNPs within genes and genes within subnetwork/pathways using ancMETA, a Bayesian graph-based framework (Chimusa and Defo, 2022), for the derived common factor (TLNWL, ESH, SA, MDD, ADHD, and AUD). AncMETA uses a Bayesian posterior probability approach that extracts common SNPs, combines the results into known biological protein-protein network datasets, performs the meta-analysis at gene and sub-network level and identifies the most significant subnetwork hubs to understand the biological pathways (Chimusa and Defo, 2022). Common SNPs (*n* = 6,870,289) were extracted from all studies and mapped to genes located within or less than 20 kb distance up/downstream of the protein-coding gene using FUMA (Watanabe et al., 2017), and were included as potential candidate genes for ancMETA analysis. Input SNPs were mapped to 16,530 protein-coding genes at gene level, of which 2,951 genes were considered to have a fixed effect, meaning the effect of each gene is assumed to be shared equally across all six traits. The genome-wide significant threshold for the gene-based test was determined to be *p* = 0.05/16,530 = 3.02 × 10^−6^. At sub-network level, ancMETA identified 693 significant hub genes, of which 98 genes had a fixed effect. The genome-wide significant threshold was determined to be *p* = 0.05/693 = 7.22 × 10^−5^. We applied the most recent version of the human protein to protein interactions (PPI) network from the IntAct database (IntAct release 239) (Kerrien et al., 2012). We performed pathway enrichment analysis on the subnetwork genes based on gene ontology (GO) and KEGG and Reactome pathways and visualised the PPI network using the Cytoscape version 3.7.2, (Shannon et al., 2003), plug-in StringApp (Doncheva et al., 2019). GO included the enrichment of subnetwork hub genes in terms of molecular function, biological process and cellular component. A *p*-value of <0.05 statistical significance was set as an enrichment standard to determine the biological importance of hub genes. We identified drug-gene interactions through the Drug-Gene Interaction Database v4.0 (DGIdb 4.0) (Freshour et al., 2020), an open access database and a web interface (www.dgidb.org). DGIdb collects data on drug-gene interaction and druggable genes from 30 different sources and 22 databases (Freshour et al., 2020). We determined the second level classification (therapeutic subgroup) of each drug using the anatomical therapeutic chemical (ATC) classification from the World Health Organisation Collaborating Centre for Drug Statistics Methodology (https://www.whocc.no/atc_ddd_index/). We visualised the interaction between the genes significantly associated with the common factor and each therapeutic subgroup using the R package circlize v0.4.15 (Gu et al., 2014).

### 2.5 Mendelian randomisation

We performed Mendelian randomisation to determine if the genetic correlations between the modifiable risk factors and suicidal behaviour arise from genes with pleiotropic effects and biological influences across the traits, or if the effects are causal. Mendelian randomisation uses genetic variants as a proxy for modifiable risk factors (an exposure) to estimate the causal effect on the outcome (Smith and Ebrahim, 2004). The principles of Mendelian randomisation can be applied to overcome bias by estimating the effect between the risk factor and outcome, in the absence of unmeasured confounders. However, the validity of Mendelian randomisation analysis is dependent on three assumptions: i) the instrument variable (genetic variant) should be associated with the exposure, ii) the instrument variable is independent of the outcome, conditional on the exposure and iii) the instrument variable is not associated with the unmeasured confounder (Burgess and Small, 2016). We used the twoSampleMR (Hemani et al., 2018), MRcML (Xue et al., 2021) and MR-APSS (Hu et al., 2022) packages in R to assess the potential causal effect of cigarettes smoked per day, alcoholic drinks per week, household income and educational achievement (school years) on suicidal behaviour risk where the cross-trait genetic correlation Bonferroni *p*-value > the corrected threshold of 9.615 × 10^−4^. For instrument variables (IV), we used the GWAS for the behavioural and socioeconomic-related traits listed in Table 1. We used the inverse-variance weighted (IVW) method with a multiplicative random effects model as the primary method and the weighted median, MR-Egger and RadialMR methods as sensitivity analyses and to detect pleiotropy. An MR-Egger intercept test of *p* > 0.05, indicates no evidence of directional pleiotropy. We used heterogeneity markers (Cochran Q-derived *p* < 0.05) from the IVW approach to represent potential horizontal pleiotropy. We applied RadialMR to detect potential outliers and removed the outliers to re-estimate the exposure-SB relationship. Genome-wide significant SNPs were selected at p < 5 × 10^−8^ significance and were clumped to ensure independence at linkage disequilibrium (LD) *r* ^2^ = 0.001 and distance of 10,000 kb. If an SNP from the instrument was unavailable in the outcome, an attempt to find proxies was made with a minimum LD *r* ^2^ = 0.8 and palindromic SNPs were aligned with minor allele frequency <0.3. Additional sensitivity analyses were performed using the constrained maximum likelihood and model averaging and Bayesian Information Criterion (cML-MA-BIC) method (Xue et al., 2021) and the Mendelian Randomisation Accounting for Pleiotropy and Sample Structure simultaneously (MR-APSS) approach (Hu et al., 2022). The cML-MA-BIC method accounts for correlated and uncorrelated horizontal pleiotropy and addresses potential violation of instrument variable assumptions identifying invalid instruments. If the goodness of fit *p*-value was >0.05, we applied the cML-MA-BIC method, otherwise the cML-MA-BIC-DP (data perturbation) method was applied. In addition to assessing horizontal pleitropy, the MR-APSS approach accounts for sample structure simultaneously and allows the inclusion of more genetic variants with moderate effects as instrument variables to improve statistical power without inflating type I errors (Hu et al., 2022). For MR-APSS, we applied its default instrument variable threshold of 5 × 10^−5^, while a threshold of 5 × 10^−8^ was applied for IVW, weighted median, MR Egger and cML-MA-BIC. The relationship between household income and TLNWL and ESH was not tested due to sample overlap as the three datasets were obtained from the UKBiobank cohort, and may introduce biased estimates. Reported estimates were converted to odds ratios where the outcome was binary, and interpreted using a conservative *p*-value threshold (0.05/number of factors with available summary statistics = 0.0083).

## 3 Results

### 3.1 SNP-based heritability

We found significant SNP-based heritability estimates of SB traits among European populations ranged from 0.0129 ± 0.0038 (1.3%) for Self-harm needing hospital treatment to 0.1461 ± 0.0892 (14.6%) for Ever attempted suicide (ESH), and 0.078 ± 0.0303 (7.8%) for completed suicide for East Asian populations (Table 2).

### 3.2 Genetic correlation between suicidal behaviour (SB) and psychiatric, behavioural, anthropometric and socioeconomic-related traits

**FIGURE 1:**
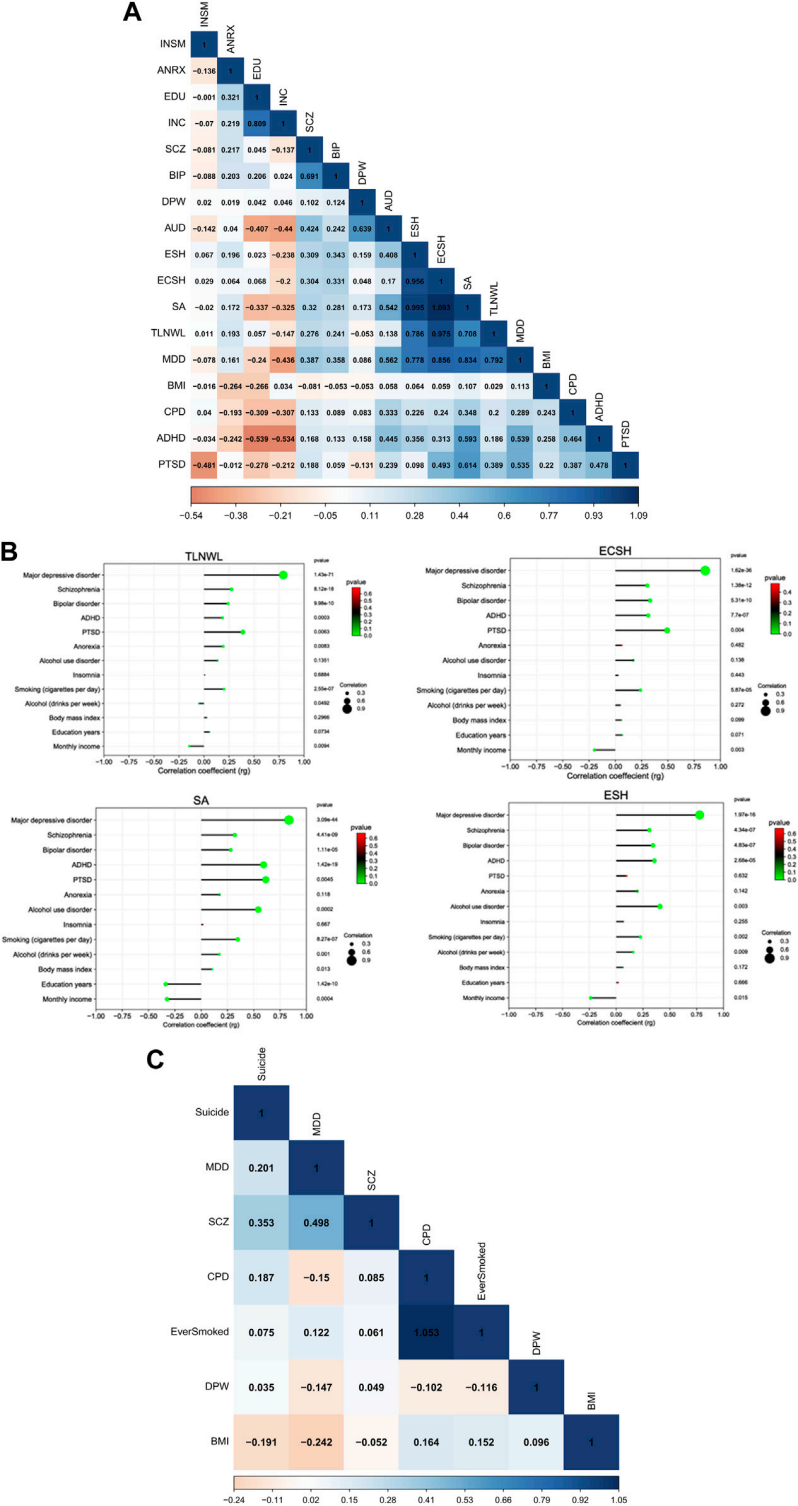
Pairwise LDSC-estimated genetic correlation between SB and psychiatric disorders, behavioural and SES traits **(A)** Heatmap showing the correlation between 17 traits among European populations, **(B)** a correlation dot plot showing the association between four suicidal behaviour traits (TLNWL, ECSH, SA and ESH)and psychiatric disorders, behavioural and socioeconomic (SES)-related traits among European populations. The size of the dot represents the strength of the correlation, and **(C)** a heatmap of seven traits including completed suicide among East Asian populations. The strength of the genetic correlation is presented as a heat scale on the *x*-axis with blue colour indicating positive and red colour representing negative correlations. Light colours represent lower correlation estimates, whereas darker colours indicate stronger correlations. Abbreviations: (SB traits) TLNWL = Thought life is not worth living, ECSH = Ever contemplated Self-harm, ESH = Ever self-harmed, SA = Suicide attempt; (Psychiatric, behavioural and socioeconomic-related traits) MDD = major depression disorder, SCZ = schizophrenia, BIP = bipolar disorder, AUD = alcohol use disorder, ADHD = attention deficit hyperactivity disorder, PTSD = post-traumatic stress disorder, ANRX = anorexia nervosa, INSM = insomnia, BMI = body mass index, DPW = drinks per week, CPD = cigarettes per day, INC = monthly income and EDU = education in years.

We used cross-trait LD Score regression (LDSC) to estimate genetic correlations among suicidal behaviour (SB), psychiatric disorders and socioeconomic-related traits among populations of European ancestry. We observed strong positive and significant correlations within the SB traits [average genetic correlation (rg) = 0.92, range, 0.71–1.09], (Figure 1A). This means that genetic factors that increase the risk of suicidal ideation, also increase the risk of attempt and self-harm. The genetic correlations were strongest between suicide attempt (SA) and ECSH [rg = 1.09 ± standard error (SE) 0.14, *p* = 1.049 × 10^−15^] and between SA and ESH (rg = 0.99 ± 0.16, *p* = 1.027 × 10^−9^) and slightly lower between SA and TLNWL (rg = 0.71 ± 0.09, *p* = 2.382 × 10^−26^). As expected, suicidal ideation phenotypes (TLNWL and ECSH) were highly correlated (rg = 0.97 ± 0.03, *p* = 2.52 × 10^−127^).

After multiple testing correction (*p* = 0.05/52 = 0.000962), five psychiatric disorders, smoking and drinking habits, and education and monthly income were significantly genetically correlated with four SB traits among European populations (Supplementary Table S1; Figure 1B). The strongest correlation with the SB traits was MDD and ECSH (rg = 0.86 ± 0.07, *p* = 1.62 × 10^−36^). Moderate positive and significant genetic correlations were observed between schizophrenia and ECSH (rg = 0.30 ± 0.04, *p* = 1.39 × 10^−12^). Similarly, moderate positive genetic correlations were observed for bipolar disorder and ESH (rg = 0.34 ± 0.07, *p* = 1.11 × 10^−5^), ADHD and attempted suicide (SA) (rg = 0.59 ± 0.07, *p* = 1.41 × 10^−19^), and AUD and SA (rg = 0.54 ± 0.14, *p* = 0.0002).

Among the behavioural traits, the strongest genetic correlations were observed for smoking habits and SA (rg = 0.35 ± 0.07, *p* = 8.27 × 10^−7^), and alcohol drinking habits and SA (rg = 0.17 ± 0.05, *p* = 0.0014). In contrast, education achievement (rg = −0.34 ± 0.05, *p* = 1.41 × 10^−10^) and monthly income (rg = −0.33 ± 0.09, *p* = 0.0004) were negatively associated with attempted suicide, meaning that educational achievement and household monthly income were protective against suicide attempt.

Among East Asian populations (Figure 1C), completed suicide was moderately correlated with schizophrenia (rg = 0.35 ± 0.13, *p* = 0.0067). We observed no significant associations between completed suicide and MDD, drinking and smoking habits and BMI.

### 3.3 Genomic structural equation modelling

**FIGURE 2:**
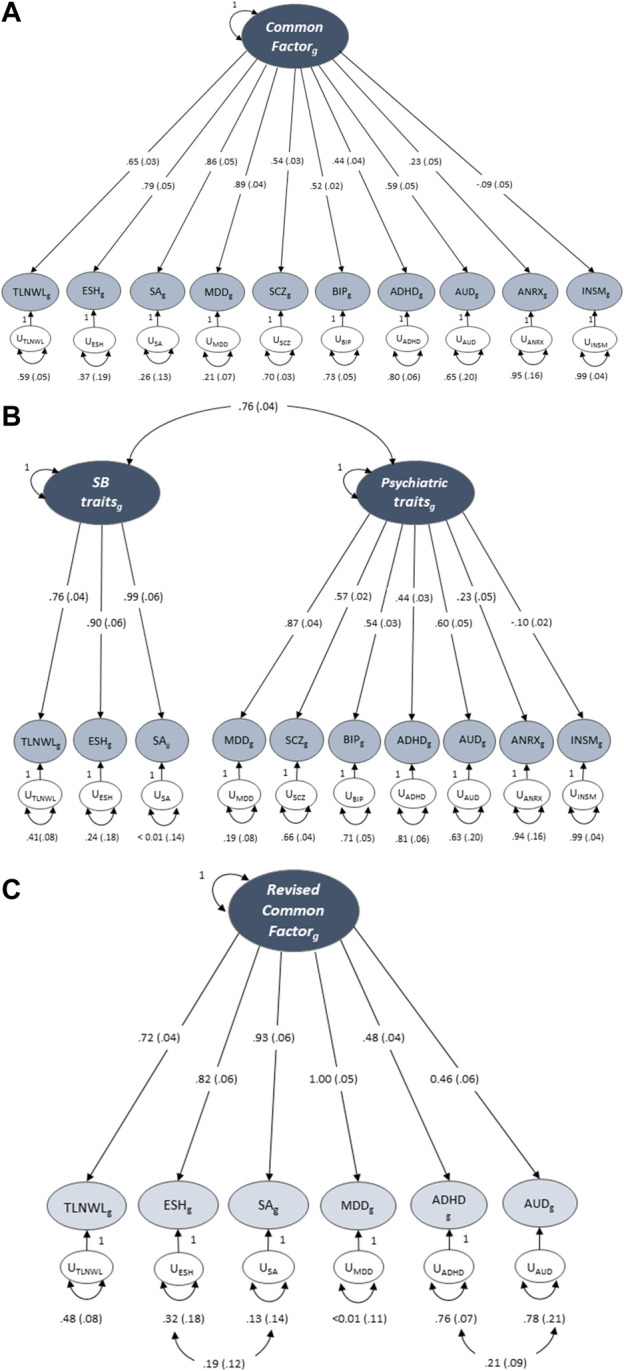
Genomic structural equation models of the standardised solutions of the SB and psychiatric traits. **(A)** Displays the path diagram (with standard errors in parenthesis) for the common factor model where there is only one factor that depicts the overarching common variance between all included traits. **(B)** Displays a two-factor model with three SB traits loaded onto the latent suicidal behaviour factor and seven psychiatric traits loaded onto a psychiatric latent factor. **(C)** Displays the path diagram for the revised common factor model which shows the factor loadings for traits that displayed a loading ≥0.40 at EFA. SCZ, BIP, ANRX and INSM did not meet the factor loading threshold.

First, we tested a model in which three SB traits (TLNWL, ESH and SA) and seven psychiatric traits (MDD, SCZ, BIP, AUD, ADHD, ANRX and INSM) loaded onto a single common latent factor (Table 3; Figure 2A). Model fit was fair for the common factor model in which the loadings were freely estimated (chi-square, X^2^(35) = 794.66, AIC = 834.66, CFI = 0.753, SRMR = 0.127). Standardised loadings indicated that MDD and SA loaded most strongly onto the common factor, while anorexia nervosa and insomnia loaded the weakest. We then assessed the fit of a correlated two-factor model where three suicidal behaviour traits were loaded onto the latent suicidal behaviour factor and seven psychiatric traits loaded onto a psychiatric latent factor (Figure 2B). We observed a strong correlation between the two latent factors (rg = 0.77 ± 0.04), however, the model fit remained suboptimal [X^2^(34) = 747.07, AIC = 789.07, CFI = 0.768, SRMR = 0.125].

**TABLE 3:** Model fit statistics for each of the SEM models performed.

Model	χ^2^ statistic	df	*p*-value	AIC	CFI	SRMR
Common factor model (10 indicators)	794.66	35	2.59 × 10^−144^	834.66	0.753	0.127
Correlated two-factor model (10 indicators)	742.48	34	3.84 × 10^−134^	784.48	0.770	0.119
Confirmatory factor analysis (CFA)						
One-factor solution (6 indicators)	51.31	8	2.28 × 10^−8^	77.31	0.964	0.086
Two-factor solution (5 indicators)	32.97	5	3.82 × 10^−6^	52.97	0.965	0.086
Three-factor solution (7 indicators)	Model did not converge					
Revised common factor model (6 indicators)	47.19	7	5.11 × 10^−8^	75.19	0.954	0.083

χ^2^—chi-squared statistic, df—degrees of freedom, AIC, Akaike’s Information Criterion, CFI, Comparative Fit Index, SRMR, standardised root mean squared residual. Good fit is indicated by CFI >0.90 and SRMR <0.85.

Next, we conducted an exploratory factor analysis and examined different factor structures that would fit the data best (Supplementary Table S2). While both the one- (Figure 2A) and two-factor (Figure 2B) CFA solutions of the 10-items fit the data adequately, the second latent factor of the two-factor solution was underspecified and explained only 11.2% of the variance. We then specified factor loadings to ≥0.4, decreasing the 10 items to six. The modified one factor model now had strong loadings for all six indicators (traits) and explained 61.3% of the variance. To further improve model fit, we evaluated a revised common factor solution of six indicators that allowed for correlated indicator residuals between ADHD and AUD and between ESH and SA (Figure 2C). This model fit the data best across all model specifications [X^2^(7) = 51.31, AIC = 75.15, CFI = 0.954 and SRMR = 0.083], suggesting that this model may represent a common or shared genetic pathway/s to suicidal behaviour across MDD, ADHD and AUD.

We extended genomic SEM to determine the genetic correlations between the revised common factor model [that represents SB (suicidal ideation, attempt and self-harm) and psychiatric disorders (MDD, ADHD and AUD)] and selected socioeconomic and behavioural traits. The revised common factor had a moderate positive correlation with smoking (rg = 0.47 ± 0.03) and inverse correlations with monthly income (rg = −0.52 ± 0.04) and education achievement (rg = −0.37 ± 0.02). Weak positive correlations were observed between the common factor and drinks per week (rg = 0.18 ± 0.02) and BMI (rg = 0.19 ± 0.02). In other words, the genetic factors that increase smoking and drinking habits, i.e., the number of cigarettes per day and drinks per week also increase SB/psychiatric disorders. In contrast, the genetic factors that influence an increase in education years and monthly income also decrease SB/psychiatric disorders; meaning higher education and household monthly income, a proxy for socioeconomic status is protective for non-fatal SB, MDD, ADHD and AUD.

### 3.4 Genes and pathways associated with the derived common factor

**FIGURE 3:**
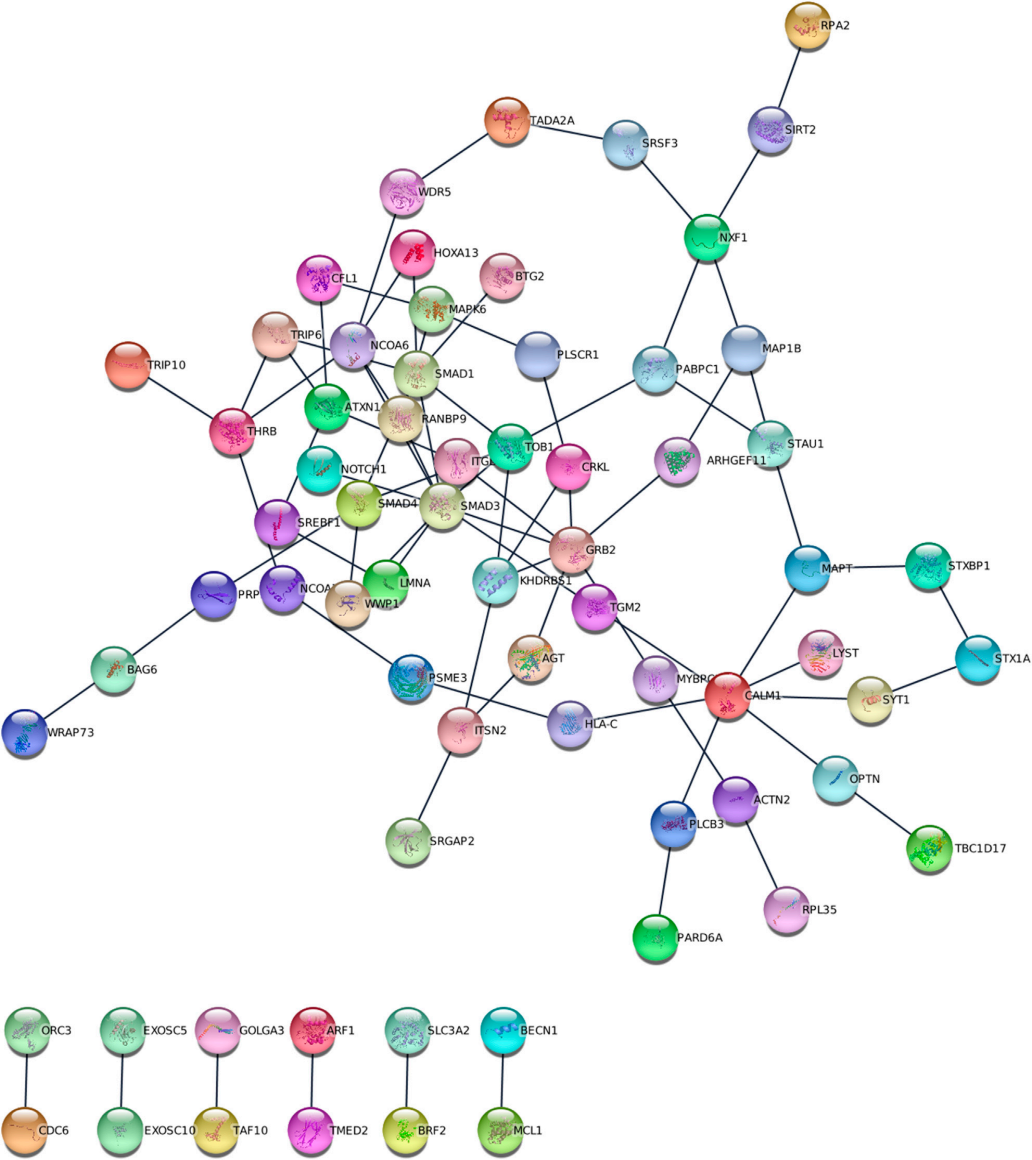
Visualisation of the 98 sub-network genes associated with the derived common factor.

ancMETA was used to perform gene and pathway-specific meta-analysis and estimate the aggregated genetic effects and the level of significance of the derived common factor (TLNWL, ESH, MDD, ADHD and AUD) on 16,530 genes. This technique identified 2,951 genes that were associated (*p* < 3.02 × 10^−6^) with the common factor at gene-level (Table 4; Supplementary Table S3) and 98 genes at sub-network level (*p* < 7.22 × 10^−5^, Supplementary Table S4; Figure 3). At gene-level, the most significant gene (*p* = 2.43 × 10^−43^) associated with the common factor was GDNF Family Receptor Alpha 3 (*GFRA3*), located on chromosome 5 and is involved in RAF/MAP kinase cascade pathway and nervous system development (Gaudet et al., 2011). Genes with significant but small effects across the six traits include the developmental pluripotency associated factor 4 (*DPPA4*) located on chromosome 3, ankyrin repeat domain 46 (*ANKRD46*) located on chromosome 8, KH domain containing 3 Like (*KHDC3L*) located on chromosome 6 and neuronal olfactomedin related ER localized protein 2 (*OLFM2*), located on chromosome 19.

**TABLE 4:** Top genes identified by gene and sub-network meta-analysis of the derived common factor.

Gene	#Study	Overall *p*-value	Beta	SD	Tau square	P_TLNWL	P_ESH	P_SA	P_MDD	P_AUD	P_ADHD
Gene level											
*GFRA3*	6	2.429E-43	3.876E-05	0.0002	0	0.0041	0.4680	0.0044	0.0024	0.0090	0.0049
*DPPA4*	6	3.631E-42	0.0004	0.0003	0	0.0010	0.4054	0.0035	0.0022	0.0756	0.0035
*ANKRD46*	6	2.188E-40	−2.828E-05	9.859E-05	0	0.0042	0.3267	0.0029	0.0054	0.1199	0.0030
*KHDC3L*	6	3.769E-40	−6.219E-05	0.0002	0	0.0025	0.3985	0.0035	0.1436	0.0078	0.0056
*OLFM2*	6	6.614E-40	2.269E-05	0.0001	0	0.0091	0.3470	0.0036	0.0084	0.0172	0.0039
Subnetwork level											
*TOB1*	6	3.154E-27	−0.00149	0.00089	0	0.0583	0.4959	0.3519	0.0888	0.3263	0.3633
*RANBP9*	6	1.395E-25	0.00013	0.00019	0	0.1193	0.4667	0.3098	0.4951	0.1708	0.4679
*SRSF3*	6	1.428E-24	0.00044	0.00017	0	0.4988	0.1240	0.2755	0.2245	0.1954	0.2748
*HSPB3*	6	1.862E-23	−0.00025	0.00025	0	0.1640	0.4588	0.4746	0.1832	0.2255	0.3668
*STK24*	6	4.081E-23	−0.00019	0.00031	0	0.1239	0.1228	0.3988	0.4963	0.1554	0.3134

SD, Standard deviation.

The most significant (*p* = 3.15 × 10^−27^) gene at sub-network level was transducer of ERBB2, 1 (*TOB1*), located on chromosome 17. In addition, the top sub-network genes were RAN binding protein 9 (*RANBP9*), located on chromosome 6, involved in developmental biology, signalling pathways and nervous system development; Serine and Arginine Rich Splicing Factor 3 *(SRSF3)*, located on chromosome 6, Heat shock protein Family B (small) member *3 (HSPB3)* located on chromosome 5, and Serine/Threonine Kinase 24 *(STK24)* on chromosome 13.

**FIGURE 4:**
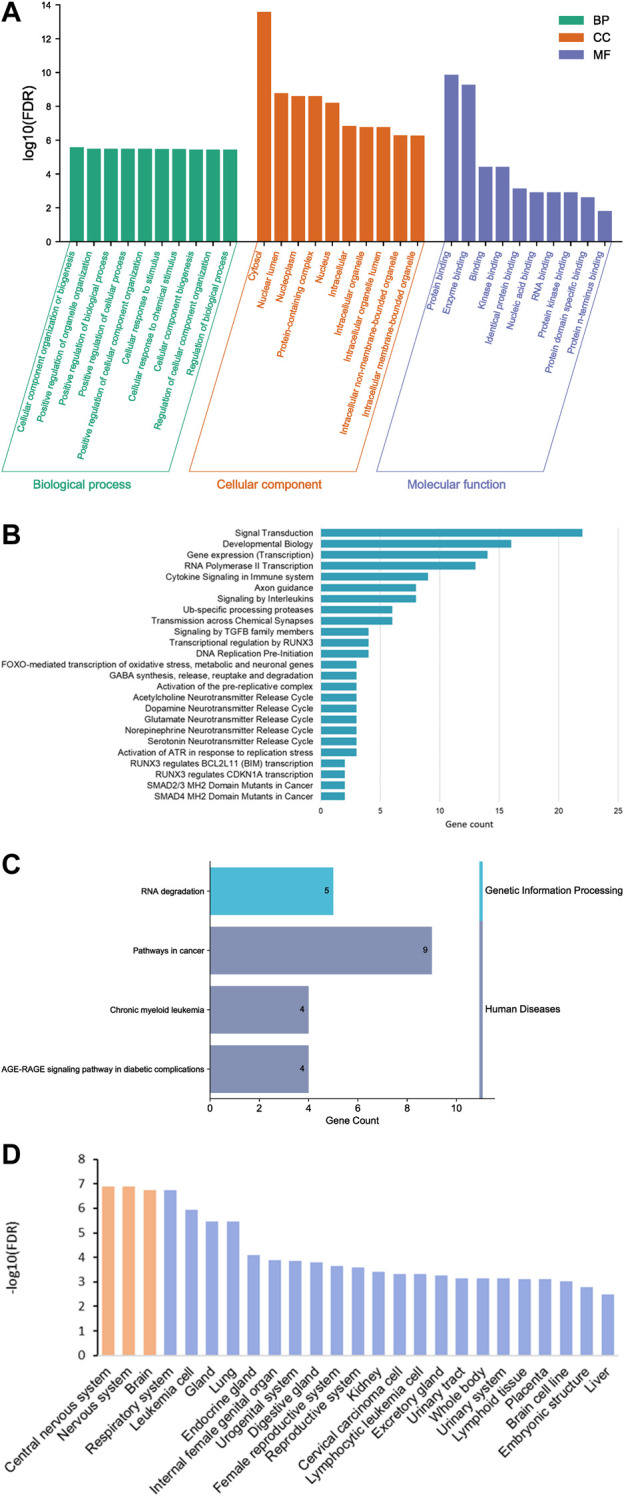
Gene ontology (GO) functional analysis histogram **(A)**, bar plot of Reactome **(B)** and KEGG pathway enrichment analysis **(C)** and enriched tissues **(D)**.

We identified 25 Reactome pathways and four KEGG pathways linking the six traits (FDR<0.05, Figure 4). KEGG pathways were related primarily with genetic information processing (RNA degradation), while Reactome pathways were related to developmental biology, particularly, nervous system development, signal transduction and gene expression (transcription). Together with two Reactome pathways (SMAD4 MH2 Domain Mutants in Cancer and SMAD2/3 MH2 Domain Mutants in Cancer), two KEGG pathways were related to pathways in cancer. Sub-network (hub) genes were mostly involved in developmental biology (Reactome pathway, FDR = 0.018), signal transduction (Reactome pathway, FDR = 0.047) and RNA degradation (KEGG pathway, FDR = 0.0028). We observed that *SMAD3* and *SMAD4* (SMAD family member 3 and 4) appeared in most enrichment pathways (Figure 3). In Gene Ontology (GO), we identified 373 categories jointly associated with the common factor: 322 biological processes, 32 cellular components and 19 molecular functions. GO enrichment analysis showed that the sub-network genes were mainly located in the cytosol (FDR = 2.6 × 10^−14^) and nuclear lumen (FDR = 1.69 × 10^−9^). Moreover, sub-network genes were enriched in molecular functions relating to protein binding (FDR = 1.37 × 10^−10^) and enzyme binding (FDR = 5.23 × 10^−10^). Likewise, cellular component organisation or biogenesis (FDR = 2.63 × 10^−6^) was identified as the most significant biological process. Most of the subnetwork genes were highly expressed in the central nervous system (FDR = 1.27 × 10^−7^), nervous system (FDR = 1.27 × 10^−7^), and the brain (FDR = 1.79 × 10^−7^, Figure 4D).

**FIGURE 5:**
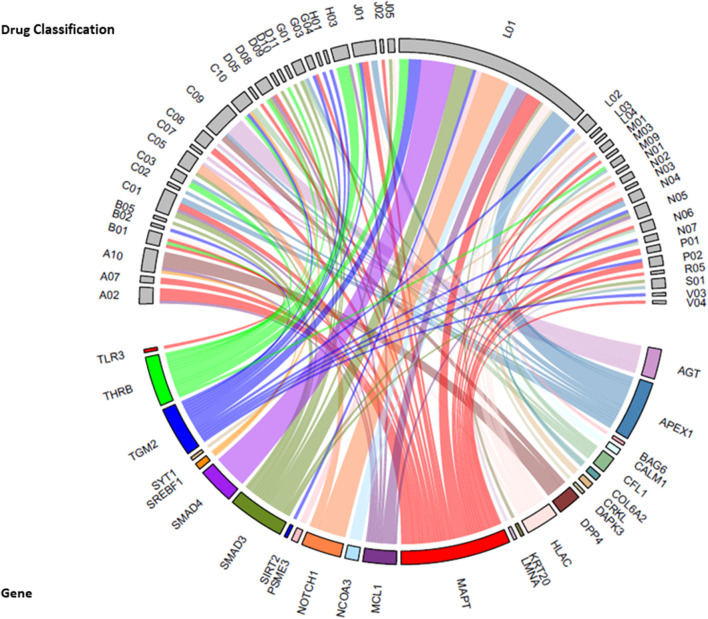
Chord diagram of sub-network genes associated with the common factor and the drug-gene interactions with the second level Anatomical Therapeutic Chemical (ATC) classification (therapeutic subgroup) of drugs. The width of each line represents the number of drugs known to interact with each gene. Therapeutic subgroup ATC Drug classifications: A02 = Drugs for acid-related disorders, A07 = antidiarrheals, intestinal anti-inflammatory/anti-infective agents, A10 = drugs used in diabetes, B01 = antithrombotic agents, B02 = antihemorrhagics, B05 = blood substitutes and perfusion solutions, C01 = cardiac therapy, C02 = antihypertensives, C03 = diuretics, C05 = vasoprotectives, C07 = beta blocker agents, C08 = calcium channel blockers, C09 = agents acting on renin-angiotensin system, C10 = lipid modifying agents, D05 = antipsoriatics, D08 = antiseptics and disinfectants, D09 = medicated dressing, D10 = anti-acne preparations, D11 = other dermatological preparations, G01 = gynecological anti-infective and antiseptics, G03 = sex-hormones and modulators of the genital system, G04 = urological, H01 = pituitary and hypothalamic hormones and analogues, H03 = thyroid therapy, J01 = antibacterial for systemic use, J02 = antimycotics for systemic use, J05 = antivirals for systemic use, L01 = antineoplastic agent, L02 = endocrine therapy, L03 = immunostimulants, L04 = immunosuppressants, M01 = anti-inflammatory and antirheumatic products, M03 = muscle relaxants, M09 = other drugs for disorders of the Musculo-skeletal system, N01 = anesthetics, N02 = analgesics, N03 = antiepileptics, N04 = anti-Parkinson drugs, N05 = psycholeptics, N06 = psychoanaleptics, N07 = other nervous system drugs, P01 = antiprotozoals, P02 = anthelmintics, R05 = cough and cold preparations, S01 = opthalmologicals, V03 = all other therapeutic products, V04 = therapeutics radiopharmaceuticals.

Drug-gene interactions: We explored potential drug-target genes among the 98 sub-network genes significantly associated (*p* = 7.22 × 10^−5^) with the common factor, for known drug interactions in the Drug Gene Interaction Database v4.0 (DGIdb 4.0) (Freshour et al., 2020). A total of 246 interactions were identified for 26 genes and 190 drugs (Supplementary Table S5). Anatomical therapeutic chemical (ATC) classifications were available for 185 drugs that were assigned to 47 therapeutic subgroups (Figure 5). The greatest number of drug-gene interactions were observed between antineoplastic agents (L01 drug classification) and *SMAD4* (SMAD Family Member 4, *n* = 11), *NOTCH1* (Notch Receptor 1, *n* = 9) and *APEX1* (Apurinic/Apyrimidinic Endodeoxyribonuclease 1, *n* = 7). Additional interactions were observed between *APEX1* and N04, anti-Parkinson drugs and between *AGT* (Angiotensinogen) and C09, drugs acting on the renin-angiotensin system.

### 3.5 Causal effect of modifiable risk factors on suicidal behaviour

We used the genetic variants associated with suicidal behaviour and genetic variants associated with smoking, alcohol drinking, education achievement and household income to determine the unique effects of each modifiable risk factor on suicidal behaviour. Mendelian randomisation analyses showed a nominal association at the *p* < 0.05 threshold of the potential effect of smoking on the risk of a suicide attempt (OR_IVW_ 1.24, 95% CI 1.03–1.49, *p* = 0.026), and suggested no causative relationship between smoking and suicidal ideation (TLNWL, ß_IVW_ 0.017, SE 0.015, *p* = 0.263) or self-harm (ESH OR_IVW_ 1.00, 95% CI 0.99–1.01, *p* = 0.437) (Table 5; Supplementary Figure S1). The intercept from the MR Egger method for suicide attempt showed minimal indication of directional pleiotropy (*p* = 0.053), and there was evidence of substantial heterogeneity (Cochran’s Q statistic *p* = 3.53 × 10^−3^). High levels of heterogeneity in the estimated effects from each SNP are an indication of potential pleiotropic effects of some of the SNPs associated with smoking and suicide attempt. We conducted a sensitivity analysis, using a radial regression framework, and identified a variant (rs34406232) on the *EGLN2* gene, as an outlier potentially introducing bias to IVW and MR Egger estimates (Supplementary Figure S1). After removing the outlier, the estimate of cigarette smoked per day on suicide attempt remained significant (random effects model: *β*_*IVW*_ 0.27, SE = 0.08, *p* = 7.05 × 10^−3^) and the Cochran’s Q statistic for heterogeneity was 32.61 (*p* = 0.001), indicating that removing the SNP made no substantive difference to the results. In addition, the cML-MA-BIC-DP results (OR = 1.30, 95% CI 1.14%–1.47%, *p* = 4.16 × 10^−5^) and the MR-APSS method (OR = 2.596, 95% CI 1.428–4.717, *p* = 1.75 × 10^−3^) were consistent with IVW method, with significant associations observed at the *p*-value threshold of 0.0083 (Supplementary Figure S2), suggesting a potential causative relationship between cigarettes smoked per day and suicide attempt.

**TABLE 5:** Two-sample Mendelian Randomisation analysis of the effect of behavioural and socioeconomic-related traits on suicidal behaviour.

Exposure	Outcome	Method	N SNPs	OR [95% CI]/β (SE)	*p*	Directional pleiotropy		Heterogeneity	
						Intercept	*p*	Q	*p*
Cigarettes smoked per day	TLNWL^a^	MR Egger	22	−0.043 (0.020)	0.053	0.005	0.002	20.03	0.456
		IVW	22	0.017 (0.015)	0.263			32.07	0.058
		Weighted median	22	0.001 (0.016)	0.957				
		cML-MA-BIC	22	0.075 (0.026)	0.004				
		MR-APSS	165	0.289 (0.089)	1.23 × 10^−3^				
	ESH	MR Egger	22	0.989 [0.978–1.002]	0.108	0.001	0.019	24.03	0.241
		IVW	22	1.002 [0.994–1.010]	0.519			31.85	0.060
		Weighted median	22	0.997 [0.987–1.005]	0.437				
		cML-MA-BIC	22	1.002 [0.939–1.069]	0.574				
		MR-APSS	165	1.352 [1.127–1.622]	1.15 × 10^−3^				
	SA	MR Egger	14	0.711 [0.418–1.209]	0.232	0.025	0.053	32.26	1.26 × 10^−3^
		IVW	14	1.237 [1.026–1.492]	0.026			44.69	3.53 × 10^−5^
		Weighted median	14	1.224 [1.038–1.443]	0.016				
		cML-MA-BIC-DP	14	1.297 [1.145–1.468]	4.16 × 10^−5^				
		MR-APSS	165	2.596 [1.428–4.717]	1.75 × 10^−3^				
Alcoholic drinks per week	SA	MR Egger	21	1.075 [0.731–1.579]	0.718	0.004	0.349	49.57	0.0001
		IVW	21	1.233 [0.948–1.602]	0.117			51.97	0.0001
		Weighted median	21	1.097 [0.834–1.362]	0.400				
		cML-MA-BIC-DP	21	1.191 [1.005–1.414]	0.044				
		MR-APSS	231	1.359 [0.942–1.961]	0.010				
Education (school years)	SA	MR Egger	53	1.051 [0.307–3.591]	0.936	−0.003	0.738	176.32	1.03 × 10^−31^
		IVW	53	0.854 [0.685–1.065]	0.162			176.77	1.69 × 10^−15^
		Weighted median	53	0.884 [0.714–1.094]	0.259				
		cML-MA-BIC	53	0.882 [0.769–1.012]	0.073				
		MR-APSS	392	0.526 [0.381–0.727]	9.61 × 10^−5^				
Household income	SA	MR Egger	37	1.294 [0.463–3.615]	0.625	−0.016	0.106	104.24	8.35 × 10^−9^
		IVW	37	0.554 [0.437–0.704]	1.29 × 10^−5^			112.43	8.58 × 10^−10^
		Weighted median	37	0.606 [0.479–0.766]	2.85 × 10^−5^				
		cML-MA-BIC-DP	37	0.603 [0.525–0.691]	3.85 × 10^−11^				
		MR-APSS	296	0.202 [0.051–0.920]	0.038				

^a^ Associations are expressed as beta coefficients, Intercept = MR, egger intercept, Q = Cochran’s Q statistic, cML-MA-BIC, constrained maximum likelihood and model averaging and Bayesian Information Criterion method, cML-MA-BIC-DP, constrained maximum likelihood and model averaging and Bayesian Information Criterion (data perturbation) method, MR-APSS, mendelian randomisation accounting for pleiotropy and sample structure simultaneously.

We also observed a potential beneficial effect of household income level on suicide attempt, with genetically predicted higher household income (odds ratio per one standard deviation increase in household income) potentially leading to a 45% decrease in the probability of attempting suicide (OR_IVW_ 0.55, 95% CI 0.44–0.70, *p* = 1.29 × 10^−5^) (Table 5; Supplementary Figure S3). Similarly, the MR Egger intercept (*p* = 0.106) suggests directional pleiotropy was not biasing the estimate, while the Cochran’s Q statistic (*p* = 8.35 × 10^−3^) showed high levels of heterogeneity, indicating that some SNPs are pleiotropic but the average pleiotropic effect is close to zero and therefore balanced. We conducted a sensitivity analysis removing two variants (rs11665242 on the *DCC* gene and rs589914 on the *RP11-734C14.2* gene) and the effects remained constant (random effects model *β*_*IVW*_ = −0.502, SE = 0.111, *p* = 7.398 × 10^−5^). Additional sensitivity analyses showed that the cML-MA-BIC-DP method (OR 0.60, 95% CI 0.53–0.69, *p* = 3.85 × 10^−11^) yielded a similar result to IVW and weighted median methods (Table 5), while the association from MR-APSS method was nominally significant (OR 0.20, 95% CI 0.05–0.92, *p* = 0.038) (Table 5; Supplementary Figure S2). These findings show a potential inverse relationship between higher household income and suicide attempt.

Our findings did not suggest a causal relationship association between suicide attempt and alcohol drinks per week or educational achievement (school years). There was no indication of directional pleiotropy (MR Egger intercept *p* = 0.349), however, the Cochran’s Q statistic (*p* = 0.0001) showed heterogeneity between individual SNP estimates at the global level (*p* = 0.0001), suggesting that some SNPs are pleiotropic but the average pleiotropic effect is close to zero (Supplementary Figure S3). We identified SNP rs4309187 in the DRD2 gene as a potential outlier and re-estimated the model after removing the outlier and the *p*-value for Cochran’s Q statistic remained significant (*p* = 0.006).

## 4 Discussion

In the present study, we analysed summary-level data from large-scale GWAS to examine the genetic correlation between suicidal behaviour and psychiatric disorders using genomic structural equation modelling. We observed strong genetic correlations between suicidal behaviour traits and moderate to strong genetic correlations between suicidal behaviour and psychiatric disorders, particularly major depression disorder. Exploratory factor analysis of individuals of European ancestry revealed a single factor that represents a common or shared genetic pathway/s to suicidal behaviour across major depression, alcohol use disorder and ADHD. We identified 98 sub-network hub genes associated with the common factor and observed pathways enriched in developmental biology, signal transduction, gene transcription and RNA degradation. Most of the subnetwork hub genes were highly expressed in the central nervous system. We identified several drug-gene interactions, involving genes in the common or shared genetic pathways that may be worth investigating as potential targets for the prevention and treatment of MDD, alcohol use disorder, ADHD and suicidal behaviour (common factor).

The observed strong genetic correlations within the non-fatal suicidal behaviour traits suggest that suicidal ideation, self-harm and attempted suicide have a shared genetic component and provides support for the possibility that suicidal behaviour may exist on a spectrum of behaviours from thinking of suicide to acting on these thoughts (Caspi et al., 2014). However, as a separate construct, non-fatal suicidal behaviour was not genetically distinct, but rather our findings suggest an interconnected network of suicidal behaviour and major depression, ADHD and alcohol use disorder that supports established epidemiological (Turecki and Brent, 2016; Fazel and Runeson, 2020) and genomic associations (Mirkovic et al., 2016; DiBlasi et al., 2021).

We identified significant positive correlations between non-fatal suicidal behaviour and psychiatric disorders, with the strongest correlation observed for major depressive disorders in individuals of European ancestry. Genetic factors play an important role in the aetiology of psychiatric disorders, with heritability estimates from twin and family studies ranging from 32% to 79% for major depression (Sullivan et al., 2000; Smoller et al., 2019), 77%–88% for ADHD (Faraone and Larsson, 2019), 81% for schizophrenia, and 57% for substance use disorders (Sullivan et al., 2012). There is well-supported evidence that psychiatric disorders are polygenic, that many common variants with small effects contribute to an increased risk (Sullivan et al., 2018) and GWAS studies have shown significant genetic overlap between psychiatric traits (Lee et al., 2019; Lee et al., 2019). Depression is a well-known risk factor for suicidal behaviour (Malone et al., 1995) and several recent large GWAS have shown an overlap between suicide attempt and depression using genetic correlation analyses or polygenic risk scoring (PRS) (Levey et al., 2019; Mullins et al., 2019; Ruderfer et al., 2019; Strawbridge et al., 2019). Further, Mullins et al. (2019) reported PRS for major depression was associated with an increased risk of attempted suicide for individuals with major depression and schizophrenia (Mullins et al., 2019). ADHD, a neurodevelopmental disorder, has been associated with depression, schizophrenia and substance use disorder in later life (Tistarelli et al., 2020), as well as an increased risk of attempted and completed suicide (Ljung et al., 2014), suggesting common underlying risk variants contribute to these disorders. A recent meta-analysis showed alcohol use disorder increases the risk of suicidal ideation, attempt and suicide completion (Darvishi et al., 2015). Further, findings from recent GWAS studies showed that PRS of completed suicide was associated with greater alcohol use and schizophrenia (Docherty et al., 2020), while attempted suicide was genetically correlated with alcohol dependence (Mullins et al., 2022). These findings suggest that there is a component of common genetic variation that is shared between suicidal behaviour and MDD, ADHD, schizophrenia and alcohol use disorder. It is possible that cross-trait assortive mating, which is explained by individuals choosing partners with specific characteristics that have no genetic relationship, may have substantially inflated the genetic correlation estimates and biased the Mendelian randomisation results (Border et al., 2022). Assortive mating across psychiatric disorders can increase the correlation between the traits of the parents, which in turn increases the correlation between the psychiatric traits of their offspring (Nordsletten et al., 2016), and may explain the genetic comorbidity across psychiatric disorders.

We identified 98 potential sub-network (hub) genes and key pathways associated with the common factor. Among the hub genes, *TOB1*, *RANBP9*, *SRSF3*, *HSPB3* and *STK24* were among the most significant. Findings from enrichment analysis suggest that the hub genes were mainly involved in developmental biology, signal transduction, gene transcription and RNA degradation pathways. *SMAD3* and *SMAD4* genes, observed in most enrichment pathways are members of the SMAD family, and code for intracellular signal transducer proteins involved in transforming growth factor-beta (TGF-ß) signalling. The TGF-beta/SMAD signalling pathway plays an important role in neurogenesis in the hippocampus and has been implicated in the development of mood disorders and the manifestation of depression and anxiety disorders (Hiew et al., 2021). Interestingly, variants in *SMAD3* have also been linked to smoking behaviour (Justice et al., 2017).

Among the top hub genes associated with the common factor, *TOB1*, *RANBP9*, *HSPB3*, and *SRSF3* were also linked to neurodegenerative disorders, such as Alzheimer’s disease, Parkinson’s, and amyotrophic lateral sclerosis, through various pathways. The RNA degradation pathway, linked to *TOB1* as indicated by the KEGG enrichment analysis, is a critical step in the control of various biological pathways. In neurons, the non-sense-mediated RNA decay (NMD) pathway serves as a regulatory mechanism to control mRNA, and mutations in the NMD genes have been associated with neurodevelopmental disorders, such as schizophrenia and neurodegenerative disorders, such as amyotrophic lateral sclerosis (Jaffrey and Wilkinson, 2018). *TOB1*, which codes for an antiproliferative protein that targets mRNA deadenylation and decay (Hosoda et al., 2011), has previously been associated with neurodegenerative disorders (Weskamp and Barmada, 2018), such as multiple sclerosis (Gironi et al., 2016) and a *TOB1* deletion has been associated with hippocampus-mediated acute stress response in animal models (Youssef et al., 2022). The primary role of the signal transduction pathway is to regulate overall growth and behaviour. *RANBP9* has been implicated in the nervous system development pathway and the regulation of a number of signalling pathways, including the signal transduction pathway. *RANBP9* interacts with proteins involved in Alzheimer’s disease and has been associated with schizophrenia (Das et al., 2017). *HSPB3* [heat shock protein family B (small) member 3], is involved in the inhibition of the apoptosis pathway and regulates cell death by inhibiting actin polymerization. *HSPB3* has previously been linked to alcohol dependence (Kapoor et al., 2014) and neurodegenerative disorders such as Alzheimer’s and Parkinson’s disease (Vendredy et al., 2020). *SRSF3* (serine and arginine-rich splicing factor 3) plays a key role in the metabolism of RNA/gene transcription (Watanuki et al., 2008). Abnormal expression of *SRSF3* can lead to aberrant gene splicing and the development of neurodegenerative disorders (Xiong et al., 2022). *STK24* (sterine/threonine kinase 24), promotes apoptosis in response to stress stimuli and caspase activation and can act as a regulator of axon regeneration in optic and radial nerves and is involved in programmed cell death (Mardakheh et al., 2016). *STK24* has been implicated in unipolar depression (Howard et al., 2019; Levey et al., 2019) and schizophrenia (Lam et al., 2019). It is worth noting that processes related to neurodegeneration may be due to the older age of study participants in UKBiobank from whom suicidal behaviour traits were obtained. Taken together, psychiatric and neurodegenerative disorders represent a heterogeneous group of neurological conditions and future studies investigating the shared molecular characteristics between suicidal behaviour, MDD, ADHD and alcohol use disorder should be explored in a younger target population to better understand the pathophysiological mechanisms that underlie psychiatric and neurodegenerative disorders.

We found positive genetic correlations between suicidal behaviour and modifiable risk behaviours such as smoking and average alcohol drinking per week, that are consistent with the observed increase in these behaviours among individuals with suicidal behaviour (Poorolajal and Darvishi, 2016; Polimanti et al., 2021) and are indicative of a shared genetic basis for these traits. The prevalence of tobacco smoking is known to be higher among individuals with mental health conditions compared to the general population (Prochaska et al., 2017). Further, tobacco smoking is considered an independent risk factor for suicidal behaviour; a meta-analysis showed that smokers are at higher risk of suicidal ideation (OR = 2.05, 95% CI 1.53–2.28), suicide attempt (OR = 2.84, 95% CI 1.49–4.19) and completed suicide (RR = 1.83, 95% CI 1.64–2.02) (Poorolajal and Darvishi, 2016). A causal association was found between earlier smoking initiation, lifetime smoking, depression and schizophrenia (Wootton et al., 2020). We used genetic variants associated with smoking and found nominally significant MR results, pointing to thepotential harmful effect of smoking intensity (increased cigarettes smoked per day) on suicide attempt, although findings were not consistent across all sensitivity analyses. Nevertheless, our results align with literature on the relationship between smoking and suicidal behaviour (Poorolajal and Darvishi, 2016) and merit further investigation for including smoking cessation and prevention in suicide prevention programs. The negative genetic correlations between suicidal behaviour and socioeconomic-related variables, i.e., education achievement and monthly income support previously reported associations between indicators of poverty and suicidal behaviour (Iemmi et al., 2016; Lorant et al., 2021). In addition, we found suggestive evidence for the protective effect of genetically predicted higher household income level on the risk of suicide attempt. Earlier work by Dohrenwend et al. have suggested that the high rate of mental disorders in disadvantaged populations can be explained by the social selection theory, that individuals with mental illness have a predisposition to declining socioeconomic status due to possible genetic factors, hospitalisations related to mental illness, and/or loss of work (Dohrenwend et al., 1992).

Our study findings suggest that individuals with major depression, ADHD or alcohol use disorder are at increased risk of suicidal behaviour. Understanding the shared biological mechanisms and pathways that may account for the similarities between suicidal behaviour and psychiatric disorders at the epidemiological, neuropathological, and molecular levels could provide potential avenues to treatment and prevention strategies. We found a number of interactions between the hub genes and the ATC therapeutic sub-groups. These exploratory findings, to be interpreted with caution, suggest that pharmaceutical treatments that are currently available may target the genetic component of the common factor. The most notable drug-gene interactions were observed between drugs grouped in the L01 drug classification, which comprises antineoplastic and immune-modulating agents, and *SMAD4* and *NOTCH1* genes. Additional drug-gene interactions were observed for *APEX1* (Apurinic/Apyrimidinic Endodeoxyribonuclease 1) and N04 drug classification, which comprises of anti-Parkinson drugs, and includes anticholinergic and dopaminergic agents.

Our study has limitations. First, we planned to examine the full spectrum of fatal and non-fatal suicidal behaviour in the exploratory factor analysis. However, only one of the nine publicly available genome-wide summary datasets consisted of individuals who completed suicide and the population was of East Asian ancestry. Owing to the confounding effects of ancestral variation in LD score regression, our factor analysis included only non-fatal suicidal behaviour data of individuals of European ancestry. Therefore, the findings from the genetic factor analyses relate only to non-fatal suicidal behaviour and do not include completed suicide. Second, the modest SNP-based heritability (z-scores <4) of completed suicides and psychiatric traits of East Asian populations meant that we could not explore the factor structure of these traits independently for individuals of East Asian ancestry. As most suicides in the world occur in low- and middle-income countries (WHO, 2021), the current analysis should be extended to include diverse populations, e.g., African and ad-mixed populations as sufficient data becomes available. This is crucial to understanding the link between suicidal behaviour and psychiatric traits to advance precision medicine efforts in countries and populations with mixed genetic ancestry patterns, where it is needed most. Third, four of the ten suicidal behaviour traits had low SNP-based heritability estimates and were therefore underpowered and not included in the genetic factor analyses. This reduced the number of datasets available for analysis; however, we were able to include at least one dataset that represented each of the SB phenotypes: suicidal ideation, suicide attempt, self-harm or completed suicide. Fourth, while Mendelian randomisation is less likely to be affected by confounding compared to observational studies, this method is limited by the number of instrumental variables available. In our study, the instrumental variables were adequate for the exposures but we were unable to test reverse causality due to the low number of instruments or lack of suitable variants for suicidal behaviour. Fifth, suicidal behaviour was defined either by self-reported items or cases identified by ICD-10 coding of hospital inpatient and death registries. Therefore, some misclassifications are expected in individuals who may have underreported their symptoms, which may underestimate suicidal behaviour. Sixth, there are known sex differences in the genetic influences of psychiatric disorders (Merikangas and Almasy, 2020) and sex-specific effects have also been identified in individuals with suicidal behaviour (Kia-Keating et al., 2007; Powers et al., 2020). We could not analyse our data stratified by sex, as sex-specific summary datasets for all datasets were not available. However, as larger, well-powered summary statistics become available, this could be addressed in the future. Lastly, this study is limited by the suicidal behaviour data that was publicly available. Because fatal suicidal behaviour is less common than non-fatal suicidal behaviour, there is less data available for completed suicides as GWAS studies of rare outcomes require more time and resources to obtain large sample sizes.

## 5 Conclusion

In conclusion, our study results support previous findings of genetic overlap between suicidal behaviour and psychiatric disorders. This highlights the importance of further investigation into the overlapping influences of these phenotypes with larger sample sizes and diverse ancestry. Understanding the biology reflected by the shared genes and related pathways could provide new directions in revealing shared etiologies that could help prioritise targets for suicidal behaviour for early intervention.

## Data Availability

The original contributions presented in the study are included in the article/Supplementary Material, further inquiries can be directed to the corresponding author.
