# Supplementary material for: The shared genetic architecture of suicidal behaviour and psychiatric disorders: A genomic structural equation modelling study
Source: Front Genet. 2023 Mar 7;14:1083969. doi: 10.3389/fgene.2023.1083969 (PMC10028147; doi:10.3389/fgene.2023.1083969)
Supplement: Supplementary file 2 [file Image1.pdf]

Supplementary Figures

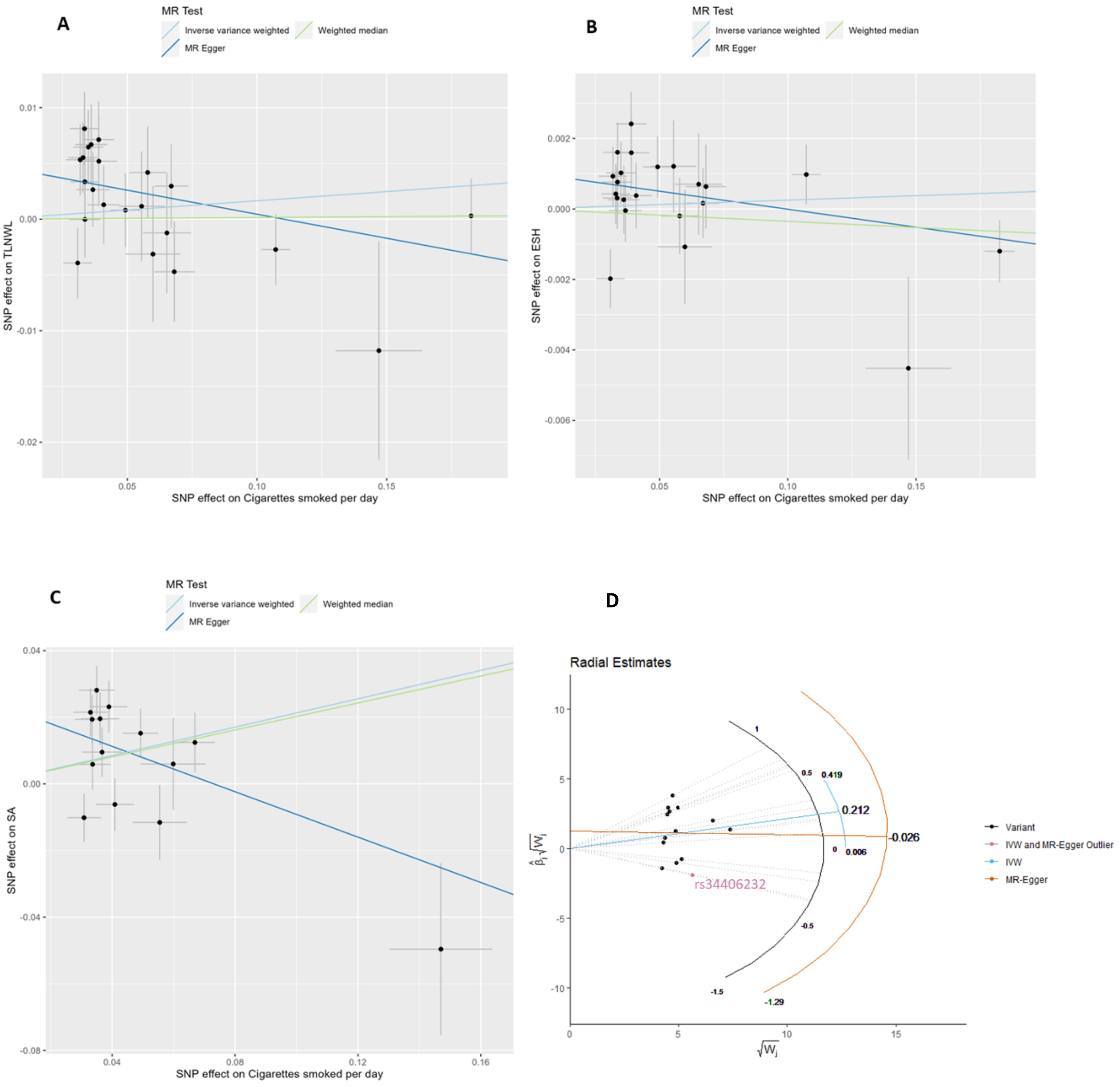

Figure S1. Scatter plots to visualize the causal effect of cigarettes smoked per day on suicidal behaviour risk. (A) TLNWL, (B) ESH and (C) SA. The Radial plot (D) of SNP-suicide attempt associations (beta*sqrt(Wj)) versus SNP-cigarette smoked per day level associations (sqrt(Wj)), with the outlier in purple.

SNP = single nucleotide polymorphism; TLNWL = Thought life is not worth living; ESH = Ever self-harmed; SA = Suicide attempt; MR = Mendelian randomization; IVW = inverse variance weighting.

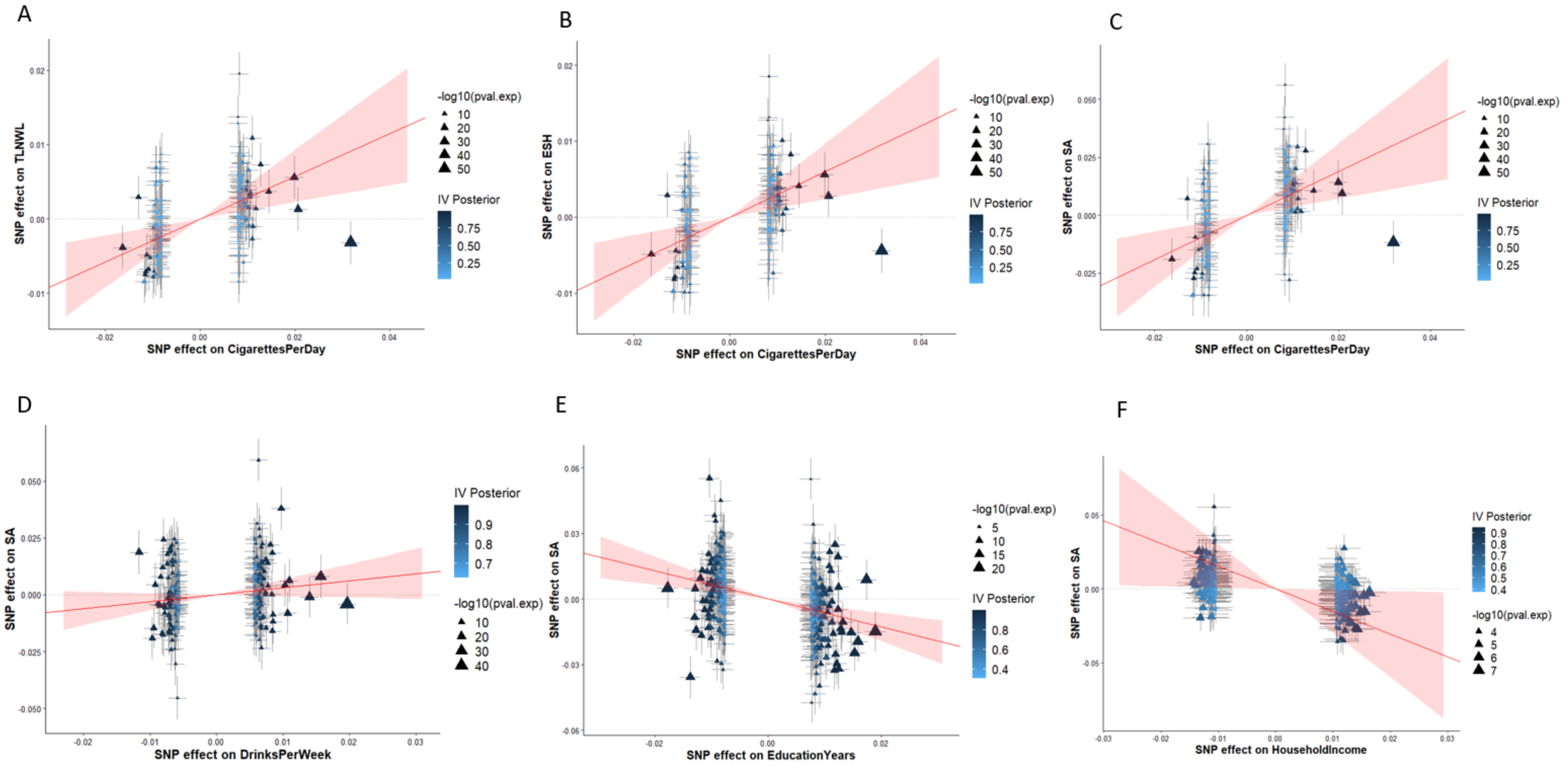

Figure S2. Visualisation of MR-APSS results using the default instrument variable (IV) threshold of $5x10^{-5}$. The estimated causal effect is indicated by a red line with 95% confidence interval indicated by a shaded area. Triangles indicate observed SNP effects with error bars. A valid instrument variable is indicated by dark blue triangle and invalid instrument by light blue triangle. SNP effects on (A) cigarettes per day and TLNWL, (B) cigarettes per day and ESH, (C) Cigarettes per day and SA, (D) Drinks per week and SA, (E) Education years and SA and (F) Household income and SA are shown.

MR-APSS = Mendelian Randomisation Accounting for Pleiotropy and Sample Structure simultaneously; SNP = single nucleotide polymorphism; TLNWL = Thought life is not worth living; ESH = Ever self-harmed and SA = Suicide attempt.

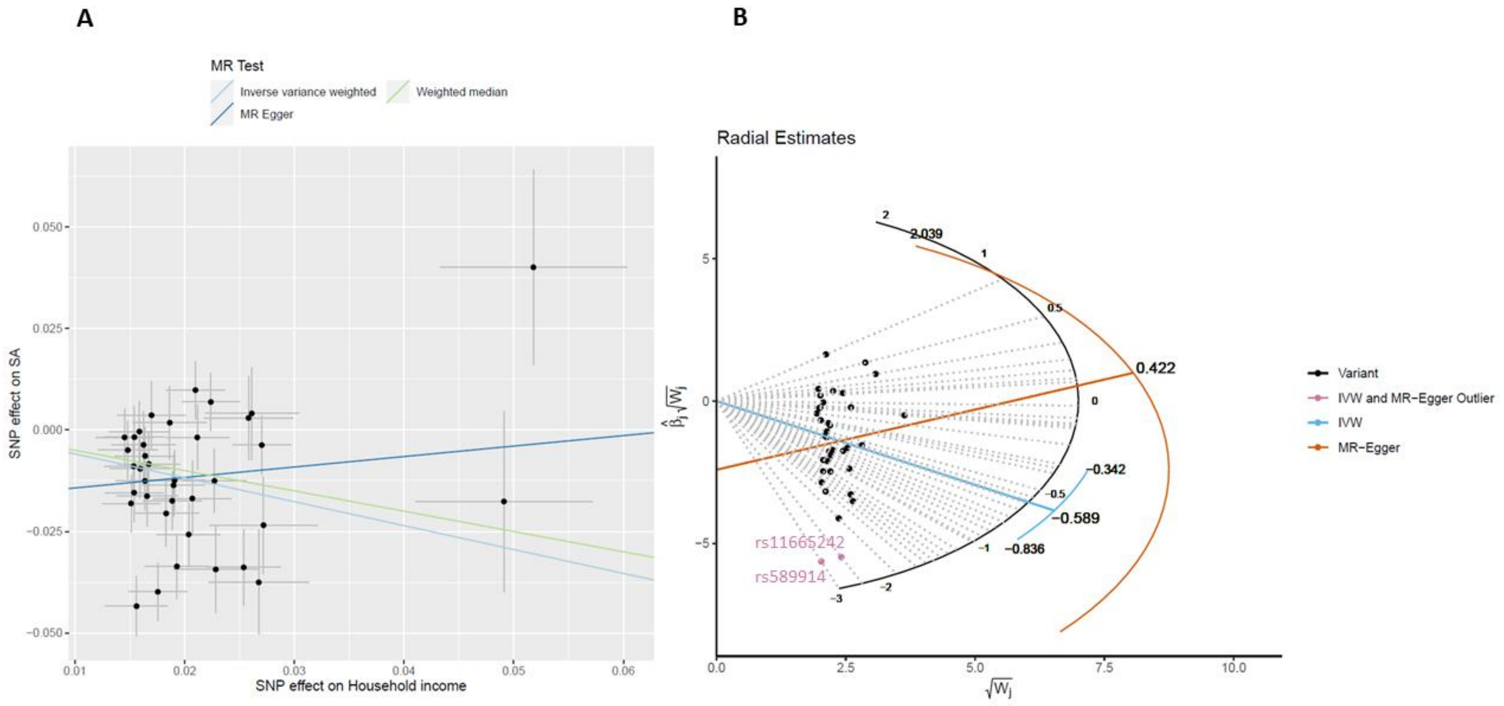

Figure S3. Scatter plot (A) and radial plot (B) of the effect of household income on risk of suicide attempt.
